# Patient Willingness‐To‐Pay and Preferences for Preimplantation Genetic Testing for Aneuploidy (PGT‐A) Provision Models in Japan

**DOI:** 10.1002/rmb2.70113

**Published:** 2026-11-10

**Authors:** Nao Wakui, Tsuyoshi Takiuchi, Junyi Shen, Mika Handa, Tadashi Kimura, Michiko Kodama, Ken‐ichirou Morishige, Tatsuyoshi Saijo

**Affiliations:** ^1^ Obstetrics and Gynecology, Graduate School of Medicine The University of Osaka Suita Osaka Japan; ^2^ Clinical Genomics, Graduate School of Medicine The University of Osaka Suita Osaka Japan; ^3^ Research Institute for Economics and Business Administration Kobe University Kobe Hyogo Japan; ^4^ Children's and Women's Health, Division of Health Science, Graduate School of Medicine The University of Osaka Suita Osaka Japan; ^5^ Sakai City Medical Center Sakai Osaka Japan; ^6^ Center for Reproductive Medicine Osaka General Medical Center Osaka Osaka Japan; ^7^ Institute for International Academic Research Kyoto University of Advanced Science Kyoto Kyoto Japan

**Keywords:** aneuploidy, health insurance, preimplantation genetic diagnosis, social values, treatment costs

## Abstract

**Purpose:**

To quantify Japanese infertile couples' willingness to pay (WTP) for preimplantation genetic testing for aneuploidy (PGT‐A) and to identify preferences for key service‐delivery attributes to inform improved models.

**Methods:**

We conducted an online survey using a discrete choice experiment among couples who participated in a PGT‐A clinical study under Japan's Advanced Medical Care B program. The primary outcome was the WTP for an additional embryo submitted. Secondary outcomes included preferences for service characteristics influencing the examination plan choice.

**Results:**

The mean WTP for an additional embryo submitted was JPY 84,072 for women and JPY 87,295 for men. Preferences were significantly influenced by the distance to the medical facility, the number of embryologists, and the duration of PGT‐A and results explanations. The mean WTP for a 1‐minute extension of the PGT‐A explanation time was JPY 7023 for women and JPY 6778 for men, while that for test‐result explanation was valued at JPY 8571 and JPY 8662, respectively.

**Conclusions:**

In Japan, infertile couples are willing to pay approximately JPY 85,000 (USD 541) per additional embryo submitted, and service components—particularly the time devoted to explaining results—substantially affect PGT‐A plan preferences. Building a delivery model based on these findings is crucial.

## 1 Introduction

Statistics on assisted reproductive technology (ART) outcomes in Japan are compiled by the Japan Society of Obstetrics and Gynecology (JSOG) and published annually. These reports indicate that in vitro fertilization (IVF) pregnancy rates decline and miscarriage rates increase after age 33 [1]. These age‐related trends are primarily attributed to the rising incidence of embryonic chromosomal abnormalities associated with maternal aging [2]. At present, PGT‐A is the only testing method capable of diagnosing embryonic chromosomal abnormalities prior to embryo transfer, and is expected to reduce miscarriage and improve pregnancy rates in patients with recurrent implantation failure, recurrent miscarriage, advanced maternal age, and chromosomal structural abnormalities [3]. In Japan, infertility treatments became covered by public health insurance in April 2022, but PGT‐A was not included in this coverage. As a self‐pay service, PGT‐A has been widely offered at facilities approved by the JSOG (244 facilities as of January 2026) despite the lack of insurance coverage for PGT‐A. Therefore, there is strong demand among patients and their families for its inclusion in public insurance coverage. To support potential medical insurance coverage for PGT‐A, a clinical study (Research Project Title: A prospective study on clinical utility of preimplantation genetic testing for aneuploidy and structural rearrangement under Advanced Medical Care B in Japan, hereinafter referred to as “the previous PGT‐A study” [4]) was initiated in April 2023 under the Advanced Medical Care B program, with the University of Osaka Hospital serving as the central research institution.

To date, PGT‐A has been priced independently by each medical facility. However, to reimburse PGT‐A at a uniform national fee under public health insurance coverage, it will become necessary to define and, where possible, standardize facility requirements and service delivery systems to help manage costs. This requires establishing a medical service framework where the insurance reimbursement level aligns with patients' financial values while maintaining provider financial sustainability. Accordingly, understanding what patients and their partners value in service delivery is critically important. In recent years, there has been a growing momentum calling for the participation of patients and citizens in healthcare decision‐making. In reproductive health in particular, where social, religious, and economic factors may exert significant influence, the need to actively involve women as stakeholders is widely recognized [5, 6]. Previous studies on preferences regarding infertility treatment indicate that while live birth rate is generally the most important treatment outcome for patients, they tend to accept a certain degree of reduction in this rate in order to improve other aspects (such as fewer adverse events, medication convenience, ease of medical visits, appropriate information provision from healthcare providers, and continuity of care with the same staff) [7, 8]. Moreover, cost burdens and cost‐sensitivity vary substantially across countries. For example, it has been reported that ART patients in China exhibit low cost‐sensitivity [7]. Considering Japan's distinctive universal health insurance system, analyzing Japan‐specific preferences regarding PGT‐A service delivery among Japanese patients is essential for discussing a future PGT‐A provision framework in Japan.

In the healthcare field, willingness‐to‐pay (WTP) is typically assessed using the contingent valuation method (CVM) or the discrete choice experiment (DCE) [9]. Because the DCE requires respondents to repeatedly choose among multiple alternatives that vary in their attributes (including price), individuals naturally engage in a comparative evaluation process in which they trade off various attributes together with the associated payment amounts. This repeated choice framework encourages respondents to consider the relative desirability of each alternative, thereby eliciting preferences that more closely resemble actual decision‐making behavior. In contrast, the CVM typically relies on a single, direct statement of WTP. Such direct elicitation formats are more vulnerable to well‐known sources of bias, including strategic responses (e.g., intentionally overstating or understating WTP), and anchoring effects in which responses are disproportionately influenced by the initially presented payment amount. Moreover, DCEs enable simple internal validity and consistency checks, such as verifying whether the probability of choosing an alternative decreases as its price increases, thereby allowing researchers to assess the rationality of respondents' choices. For these reasons, and to obtain WTP estimates that are more behaviorally grounded and internally consistent, this study adopted a DCE to elicit WTP.

This study aimed to quantify the price range and healthcare system requirements (e.g., access to medical facilities, facility characteristics, and healthcare provision systems) valued by Japanese patients and their partners regarding PGT‐A, and to examine how these preferences are influenced by their sociodemographic characteristics. Using WTP as a monetary metric, we estimated attribute‐level WTP to compare the relative value placed on different elements of the PGT‐A service‐delivery system using a common scale [10, 11]. These findings may inform the design of a patient‐centered PGT‐A provision system under public insurance coverage in Japan. No previous reports in Japan have evaluated PGT‐A from an economic perspective; therefore, these results provide policy‐relevant insights for reproductive healthcare delivery.

## 2 Methods

### 2.1 Study Design and Participants

This cross‐sectional online questionnaire survey targeted couples who had consented to participate in the previous PGT‐A study (case registration: April 2023 to March 2024). Invitation letters with sex‐specific survey links were mailed to 377 couples. Due to the lack of prior literature in this population and the cognitive burden of the 10‐task DCE, we conservatively anticipated a ~30% response rate for women and slightly lower for men. Accordingly, target sample sizes were set at 100 women and 70 men; however, all received responses were analyzed to maximize precision. To prevent duplicate entries, each participant used a unique, consecutive serial code assigned for couple (e.g., 1221‐1 for the woman,1221‐2 for the man). Informed consent was obtained via a mandatory box before accessing the questionnaire. Completing all sections incentivized participants with a JPY 1000 gift voucher. Along with our facility, three other participating clinics from the previous PGT‐A study (HORAC Grand Front Osaka Clinic, IVF Osaka Clinic, and IVF Namba Clinic) mailed invitations to their respective patients. The response period was set at 1 month, and the survey targeted patients who had undergone PGT‐A between 9 and 20 months before questionnaire distribution.

### 2.2 Survey Items

The online questionnaire was sex‐specific: men completed items (1) and (4), whereas women completed items (1) through (4). Responses were collected via single‐choice dropdown menus, except for PGT‐A indications.

1. Sociodemographic characteristics: occupation, education, number of children with the current partner, and annual household income
2. History of infertility or recurrent pregnancy loss treatment: duration of total treatment and ART treatment, and number of previous oocyte retrievals and embryo transfers
3. Details regarding PGT‐A conducted in the previous PGT‐A study: indications for PGT‐A, distance from home to the facility performing PGT‐A, number of oocyte retrievals performed for PGT‐A submission, age at oocyte retrieval or sperm collection, number of embryos submitted for PGT‐A, number of category A/B embryos obtained, whether category A/B embryos were transferred, and pregnancy outcome (category A or B embryos were defined according to the JSOG “The guideline for Embryo Diagnosis in Preimplantation Genetic Testing” [12]: Appendix 1)
4. Choice Set: 10 Questions (DCEs; 10 tasks per participant):

Each choice set evaluated three hypothetical test plans based on eight attributes and their levels:

- Cost per embryo: JPY 50,000, JPY 60,000, JPY 70,000, JPY 80,000, JPY 90,000, or JPY 100,000
- Number of embryos submitted for PGT‐A: 2, 4, 6, 8, or 10
- Facility type: Small clinic (≤ 200 annual oocyte retrievals), Large clinic (> 200), or University hospital (affiliated with a university, regardless of volume)
- Distance from home to the facility performing PGT‐A: 5, 20, 50, or 100 km
- Annual number of PGT‐A cases performed at the facility: 10, 50, 100, or 200 cases
- Number of embryologists: 1, 7, 14, or 20
- Explanation time for PGT‐A: 5, 10, 20, or 30 min
- Explanation time for results of PGT‐A: 5, 10, 20, or 30 min

Participants selected their most preferred option for each of 10 choice‐set questions (Figure 1 shows an example of a choice set, and Appendix 2 details all 10 choice sets). These choice sets were generated using a D‐optimal design approach to estimate the main effects of each attribute. The 10 choice sets were arranged in a randomized order, and this identical sequence was presented to all respondents. Because a non‐choice alternative may hinder the analysis when the objective is to examine the effects of attribute levels on choice behavior [13], no opt‐out options were provided. The cost options per embryo were based on actual fees paid by patients in the JSOG multicenter study on PGT‐A (2017–2018) [14]. Other attributes levels (Facility type, distance, annual PGT‐A cases volume, and number of embryologists) were derived from published surveys of Japanese ART facilities (2021–2023) [15, 16]. The time spent explaining PGT‐A and results was set as expected in general clinical practice.

**FIGURE 1:**
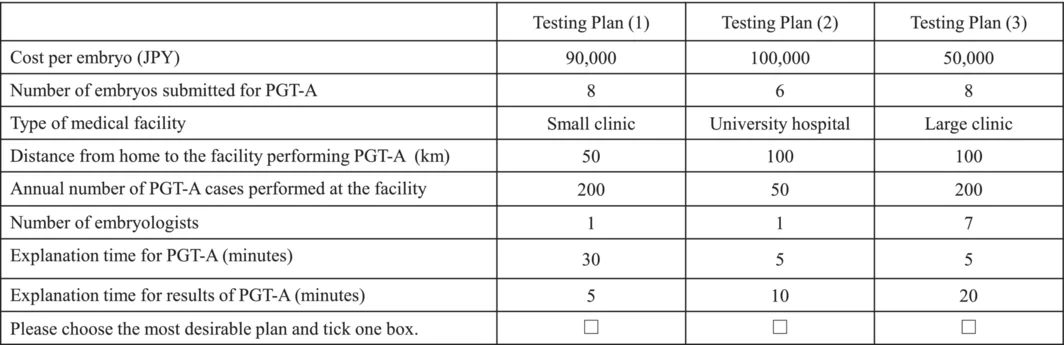
An example of a choice set used in a discrete choice experiment (DCE). Participants were presented with three hypothetical PGT‐A testing plans defined by eight attributes (cost per embryo, number of embryos submitted, facility type, distance from home, annual PGT‐A case volume, number of embryologists, explanation time for PGT‐A, and explanation time for test results) and were asked to select the most desirable plan in each task. DCE, discrete choice experiment; PGT‐A, preimplantation genetic testing for aneuploidy.

### 2.3 Evaluation Criteria

The primary outcome was the WTP for an additional embryo submitted for PGT‐A. To explore whether WTP differs by clinical status—as expectations of PGT‐A benefits may vary across treatment stages—participants were categorized into four subgroups based on their clinical outcomes: Group a (those who did not obtain category A/B embryos via PGT‐A), Group b (those who obtained category A/B embryos but were awaiting embryo transfer), Group c (those who transferred category A/B embryos but did not achieve pregnancy or experienced miscarriage), and Group d (those who transferred category A/B embryos and subsequently achieved pregnancy or a live birth).

Secondary outcomes were the preferences influencing testing plan selection based on the remaining non‐monetary attributes (facility type, distance, annual PGT‐A cases performed volume, number of embryologists, and explanation time for PGT‐A and results). We evaluated which elements patients and their partners prioritized within the testing provision system and placed monetary value on.

### 2.4 Empirical Methods

In the analysis of discrete choice experiment data, the conditional logit (CL) model is widely used as the baseline specification to investigate how respondents value the presented attributes. However, it is extremely difficult to examine heterogeneity among individuals using a CL model. One way to circumvent this difficulty is to use the estimates obtained from the latent class logit (LCL) model. The LCL model assumes that the population consists of several latent classes S and that unobserved heterogeneity among individuals can be captured by these classes by estimating a different parameter vector in the corresponding attribute matrix.

The LCL model cannot be estimated unless S (the number of classes) is given. Therefore, the central issue in the LCL model is determining S. The literature recommends several information criteria for this purpose [17, 18, 19, 20]. Among these, the Akaike Information Criterion (AIC) and Bayesian Information Criterion (BIC) based on the log likelihood at convergence with s classes, sample size, and number of parameters are popular for determining S.

An alternative approach that can account for individual heterogeneity in discrete choices is called the random parameter logit (RPL) model, which allows the parameters to vary randomly across individuals according to specified distributions (e.g., normal, log‐normal, or triangular). Under this framework, each individual is characterized by their own set of parameter values. From this perspective, the RPL model can be viewed as a special case in which every individual in the sample is effectively treated as a separate latent class—corresponding to an LCL model with N classes, where N is the sample size.

The details of the CL, LCL, and RPL models, as well as WTP calculations, are provided in Appendix 3. In this study, we estimated the responses to the ten choice sets using the CL, LCL, and RPL models. It should be noted that although the choice sets present “the cost per embryo” as an attribute, in the model estimation this attribute was converted into “the total cost,” calculated as the cost per embryo multiplied by the number of embryos submitted for testing. In a discrete choice experiment, WTP is derived by taking the ratio of an attribute's marginal utility, measured by its estimated parameter, to the marginal utility of cost, as this reflects the marginal rate of substitution between the attribute and money.

In the subgroup analysis, once individual WTP values were obtained, differences in mean WTP between groups were examined using pairwise two‐sided *t*‐tests, with the Bonferroni correction applied to adjust for multiple comparisons. In addition, in the analysis of the combined male–female sample, we investigated the extent to which each background characteristic (e.g., education, income, occupation, and number of existing children) contributed to variations in WTP.

## 3 Results

### 3.1 Patient Backgrounds

Figure 2 shows the respondent flow. Invitations were sent to 377 couples, and 214 women (Response rate: 56.8%) and 180 men (47.7%) responded via the online form. Of these, responses from 214 women and 179 men were valid, and were included in the analysis. Based on the serial codes, paired data were available for 148 couples. Appendix 4 shows the distribution of valid respondents by treatment facility. Due to errors in entering serial codes, the treatment facility could not be identified for some respondents, and some female and male responses could not be linked as couples. Among the female respondents, 204 women had actually undergone PGT‐A, and 156 of those women obtained category A/B embryos. The subgroup distribution (as defined in Section 2.3) was as follows: Group a (*n* = 58), Group b (*n* = 11), Group c (*n* = 52), and Group d (*n* = 93). Table 1 summarizes the sociodemographic characteristics. For both women and men, the most common educational attainment was a junior college or university degree. Approximately 60% of couples had no children with their current partner. Household income was most concentrated in the JPY 6–8 million range, exhibiting a normal distribution.

**FIGURE 2:**
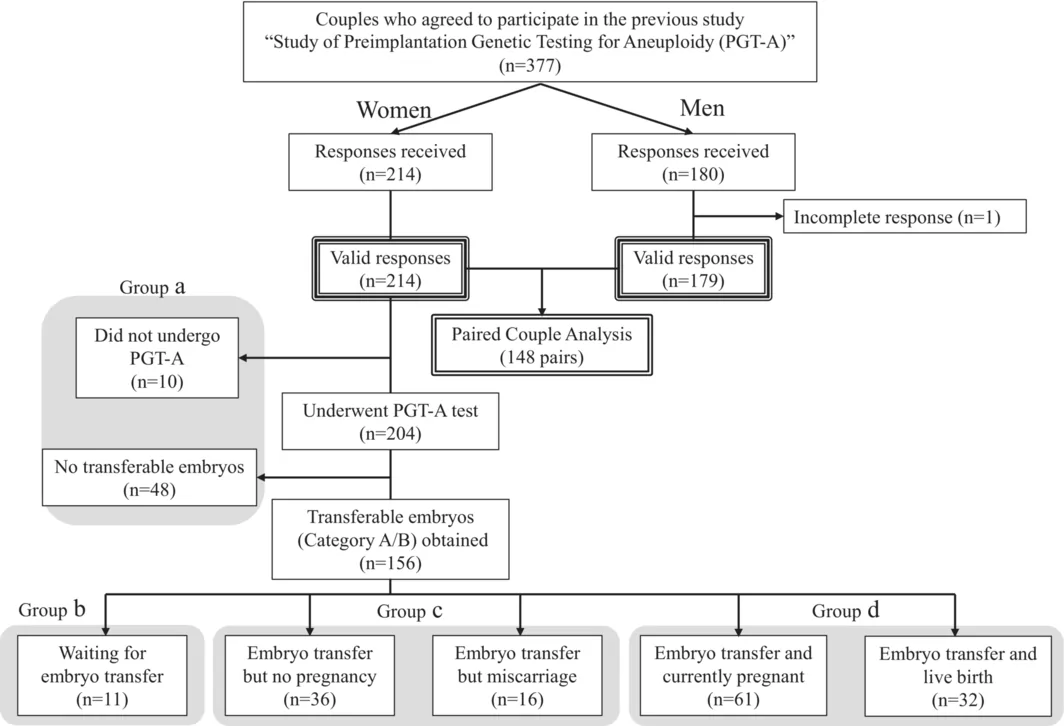
Flow diagram of study participants and subgroup classification. Invitations were mailed to 377 couples enrolled in the previous study, and responses were obtained via an online questionnaire. Valid responses were analyzed, and female respondents were further classified into Groups a–d based on PGT‐A/embryo transfer outcomes. Category A/B, embryos classified as transferable Categories A or B according to the Japan Society of Obstetrics and Gynecology criteria; PGT‐A, preimplantation genetic testing for aneuploidy.

**TABLE 1:** Sociodemographic characteristics of respondents.

Occupation (number, %)	Women (*n* = 214)	Men (*n* = 179)	Highest educational attainment (number, %)	Women (*n* = 214)	Men (*n* = 179)
Company employee	85 (39.7)	131 (73.2)	Middle School	3 (1.4)	4 (5.1)
Civil servant/teacher	14 (6.5)	28 (15.6)	High School	18 (8.4)	21 (11.7)
Self‐employed	8 (3.7)	16 (8.9)	Vocational School	40 (18.7)	17 (9.5)
Part‐time job	41 (19.2)	0 (0.0)	Junior college/University	135 (63.1)	105 (58.7)
Student	0	0 (0.0)	Graduate School	12 (5.6)	30 (16.8)
Homemaker	59 (27.6)	0 (0.0)	Currently enrolled in school	0	0 (0.0)
Others	6 (2.8)	2 (1.1)	Others	1 (0.5)	0 (0.0)
Do not wish to answer	1 (0.5)	2 (1.1)	Do not wish to answer	5 (2.3)	2 (1.1)

Number of children with current partner (number, %)	Women (*n* = 214)	Men (*n* = 179)	Household annual income (JPY) (number, %)	Women (*n* = 214)	Men (*n* = 179)
0	130 (60.7)	108 (60.3)	< 2 million	2 (0.9)	4 (2.2)
1	65 (30.4)	55 (30.7)	≥ 2, < 4 million	9 (4.2)	11 (6.1)
2	15 (7.0)	14 (7.8)	≥ 4, < 6 million	44 (20.6)	33 (18.4)
3 or more	1 (0.5)	0 (0.0)	≥ 6, < 8 million	47 (22.0)	45 (25.1)
Others	1 (0.5)	0 (0.0)	≥ 8, < 10 million	42 (19.6)	33 (18.4)
Do not wish to answer	2 (0.9)	2 (1.1)	≥ 10, < 15 million	37 (17.3)	29 (16.2)
			≥ 15 million	12 (5.6)	12 (6.7)
			Do not wish to answer	21 (9.8)	12 (6.7)

The following items were answered only by women. The most frequent durations were 2–3 years for general fertility treatment (29.0%) and 1–2 years for ART treatment (30.8%). Regarding previous embryo transfer cycles, 35.5% had undergone 5 or 6 cycles, and 23.4% had undergone 7 or more cycles (Appendix 5). Indications for PGT‐A (multiple responses allowed) included recurrent implantation failure (172/214; 80.4%), recurrent miscarriage (104/214; 48.6%), and chromosomal structural abnormalities (22/214; 10.3%), with substantial overlap observed between the first two categories. Regarding the distance from home to the facility performing PGT‐A, 58/213 women (27.2%) traveled less than 10 km, and 71/213 women (33.3%) traveled between 10 and 30 km; collectively, these two groups accounted for more than half of the total. However, at the time of the previous PGT‐A study, only four facilities nationwide, including ours in Osaka Prefecture, provided PGT‐A under Advanced Medical Care B. Consequently, 21/213 women (9.6%) traveled over 300 km for treatment (Appendix 6).

Table 2 summarizes the details regarding gamete collection and results for PGT‐A. The majority of women were between their late 30s and 43 years old at the time of oocyte retrieval, while the majority of men were in their late 30s to early 40s at the time of semen collection. In the previous PGT‐A study, participants could undergo multiple oocyte retrieval cycles to obtain embryos for submission; however, once embryos were submitted for PGT‐A within the study, re‐enrollment was not permitted even if no category A/B embryos were obtained. Consequently, 6/210 women (2.8%) ultimately submitted no embryos for testing, and among the 204 women who underwent PGT‐A testing, 48 (23.5%) did not obtain any category A/B embryos.

**TABLE 2:** Details of PGT‐A performed in the previous PGT‐A study.

Details on gametes sampling						Details of results regarding PGT‐A			
Number of oocyte retrievals for PGT‐A (number, %)		Age at time of oocyte retrieval for PGT‐A (number, %)		Age at time of sperm collection for PGT‐A (number, %)		Number of embryos submitted for PGT‐A (number, %)		Number of embryos categorized as A or B based on PGT‐A (number, %)	
0	3 (1.4)	< 20	0	< 20	0	0	6 (2.8)	0	48 (23.5)
1 or 2	199 (93.0)	≥ 20, < 25	0	≥ 20, < 25	0	1 or 2	91 (43.3)	1 or 2	95 (46.6)
3 or 4	9 (4.2)	≥ 25, < 30	11 (5.2)	≥ 25, < 30	8 (3.8)	3 or 4	42 (20.0)	3 or 4	32 (15.6)
≥ 5	3 (1.4)	≥ 30, < 35	49 (23.3)	≥ 30, < 35	41 (19.5)	5 or 6	28 (13.3)	5 or 6	19 (9.3)
		≥ 35, < 38	51 (24.3)	≥ 35, < 40	67 (31.9)	7 or 8	9 (4.3)	7 or 8	5 (2.4)
		≥ 38, < 40	47 (22.4)	≥ 40, < 45	66 (31.4)	9 or 10	12 (5.7)	≥ 9	5 (2.4)
		≥ 40, < 43	52 (24.8)	≥ 45, < 50	23 (11.0)	≥ 11	22 (10.5)		
				≥ 50	5 (2.4)				
Total	214		210^a^		210^a^	Total	210		204

*Note:* “The previous PGT‐A study” refers to a clinical study on PGT‐A that was launched in April 2023 under the Advanced Medical Care Program B, led by the University of Osaka Hospital.

Abbreviation: PGT‐A, preimplantation genetic testing for aneuploidy.

^a^ Four persons did not fill in the answers to this question.

### 3.2 WTP

Three logit models (LCL, CL, and RPL) were applied separately for women and men, calculating coefficients and their statistical significance for the choice set items (Tables 3 and Appendix 7). The optimal number of latent classes was determined using the AIC and BIC measures. We estimated models with various numbers of classes (2, 3, 4, and 5) and found that both the AIC and BIC reached their minimum values in the 2‐class model for both the women's and men's samples. In addition, in the RPL model, the coefficient for the total cost of PGT‐A testing was specified as a fixed parameter, whereas the coefficients for the other attributes were assumed to follow a normal distribution. Since the 2‐class logit (LCL) model yielded the highest predictive power, this model (Table 3) was used for subsequent analyses.

**TABLE 3:** Estimation results of the latent class logit model.

	Female		Male	
	Class I	Class II	Class I	Class II
Total PGT‐A cost (10,000 JPY)	−0.0749***	−0.0444***	−0.0539***	−0.0380***
Number of embryos submitted for PGT‐A	0.5461***	0.4142***	0.4664***	0.3328***
Distance from home to the facility performing PGT‐A (km)	−0.0609***	−0.0075***	−0.0689***	−0.0097***
Annual number of PGT‐A cases performed at the facility	−0.0002	0.0053***	−0.0098	0.0040***
Number of embryologists	0.2102***	0.0634***	0.0828**	0.0599***
Explanation time for PGT‐A (min)	0.0839***	0.0158***	0.0816***	0.0142***
Explanation time for results of PGT‐A (min)	0.0940***	0.0234***	0.1009***	0.0190***
Type of medical facility				
University hospital (vs. small clinic)	−1.3074***	0.5360***	0.6017	0.0726
Large clinic (vs. small clinic)	−0.8557*	0.3064**	1.3617	−0.0305
Probability of entering each class	0.4228	0.5772	0.2482	0.7518
Predictive power	72.99%		70.50%	
Observations	2140		1790	

*Note:* Total PGT‐A cost was calculated as the per‐embryo testing cost multiplied by the number of embryos submitted (in units of 10,000 JPY). Predictive power refers to the proportion of choices correctly predicted by the model. Standard errors and *z*‐values are omitted to save space. ***p < 0.01, **p < 0.05, *p < 0.10.

Abbreviation: PGT‐A, preimplantation genetic testing for aneuploidy.

In the tables, the total cost of PGT‐A was coded as a continuous variable and calculated as the per‐embryo testing cost multiplied by the number of embryos submitted. Number of embryos submitted for PGT‐A, distance from home to the facility performing PGT‐A, annual number of PGT‐A cases performed at the facility, number of embryologists, explanation time for PGT‐A, and explanation time for results of PGT‐A were also coded as continuous variables using the values presented in the choice sets. University hospital and large clinic were coded as dummy variables, taking a value of 1 if the medical facility was a university hospital or a large clinic, respectively, and 0 otherwise.

Preferences were largely similar between women and men. Among women, preferences centered on the number of embryologists and the thoroughness of explanations, broadly dividing them into Female‐Class I (preferring small clinics) and Female‐Class II (preferring university hospitals or large clinics). The estimated probability of belonging to Female‐Class II was higher than that of Female‐Class I (57.72% vs. 42.28%). Men were broadly categorized into Male‐Class I, who did not prioritize the annual number of PGT‐A cases performed at the facility, and Male‐Class II, who preferred facilities with a high annual procedure volume. The estimated probability of belonging to Male‐Class II was higher than that of Male‐Class I (75.18% vs. 24.82%). For both sexes across all classes, the distance from home to the facility, the number of embryologists, the explanation time for PGT‐A, and the explanation time for results were all significantly associated with respondents' preferences for their testing plan.

Using NLOGIT 6.0, we derived individual‐based WTP estimates for each attribute from the 2‐class logit model. These were calculated as the posterior‐probability‐weighted average of class‐specific WTP estimates, where the posterior class membership probabilities were obtained from the estimated class allocation model for each respondent. The mean WTP for an additional embryo submitted was JPY 84,072 (USD 535; 1 USD = 157.18 JPY as of December 25, 2024) for women, with a statistical difference between Female‐Class I (JPY 74,216 or USD 472) and Female‐Class II (JPY 91,505 or USD 582). The mean WTP for an additional embryo submitted for men was JPY 87,295 (USD 555), which was higher than that for women. After confirming via an F‐test that the women and men groups did not have equal variances (*F* = 521.2489, *p* < 0.0001), a Welch's *t*‐test revealed a statistically significant difference in the mean WTP values between women and men (*t* = −5.2721, *p* < 0.0001). The difference between Male‐Class I (JPY 86,642 or USD 551) and Male‐Class II (JPY 87,502 or USD557) was less pronounced than that observed among women (Table 4).

**TABLE 4:** Calculation of WTP for an additional embryo submitted by sex.

	2 class logit model		
	Class I	Class II	Pooled
Female data			
WTP			
Mean	74216 (USD 472)	91505 (USD 582)	84072 (USD 535)
(JPY, calculated per individual)			
SD	1,485	3,045	8,933
Minimum value	72,940	81,103	72,940
Maximum value	78,459	93,333	93,333
Male data			
WTP			
Mean	86642 (USD 551)	87502 (USD 557)	87295 (USD 555)
(JPY, calculated per individual)			
SD	123	135	391
Minimum value	86,539	86,988	86,539
Maximum value	86,957	87,573	87,573

*Note:* 1 USD = 157.18 JPY (as of December 25, 2024).

Abbreviation: WTP, willingness to pay.

In an additional analysis of the explanation time, the mean WTP values for a one‐minute extension of the explanation time for PGT‐A were JPY 7023 (USD 45, standard deviation (SD): 3343, range: 3558–11,189) for women and JPY 6778 (USD 43, SD: 4299, range: 3733–15,094) for men. Similarly, the mean WTP values for a one‐minute extension of the explanation time for the results of PGT‐A were JPY 8571 (USD 55, SD: 3183, range: 5271–12,538) for women and JPY 8662 (USD 55, SD: 5174, range: 4996–18,669) for men. For all items, there was no significant difference in the increase in WTP between women and men (explanation for PGT‐A, *t* = 0.6206, *p* = 0.5353; explanation for results, *t* = −0.2048, *p* = 0.8379.).

Next, for each individual's WTP obtained from the 2‐class logit model applied to the mixed‐sex group, ordinary least squares regression analysis revealed that among sociodemographic factors, being male (coefficient 0.2899, *p* < 0.001) was associated with a higher WTP. A tendency toward lower WTP was observed with an increasing number of existing children with the partner (coefficient −0.0933, *p* = 0.076), whereas educational attainment, occupation, and household income did not significantly contribute to WTP. For the 148 respondent pairs, the ratio of WTP per couple showed that, on average, men's WTP was 1.0548 times that of women (SD: 0.1125, range: 0.9272–1.2006).

Finally, we examined differences in WTP based on embryo transfer treatment outcomes among female respondents (Table 5). Group b (obtained category A/B embryos but awaiting transfer) demonstrated a significantly higher WTP for an additional embryo submitted (JPY 104,537 or USD 665) compared to other groups (*p* < 0.001). Furthermore, Group d (achieved pregnancy or live birth) showed a significantly higher WTP compared with Group c (failed to achieve pregnancy or experienced miscarriage) (*p* = 0.035).

**TABLE 5:** Calculation of WTP for an additional embryo submitted by treatment outcomes.

	Sub‐groups (number)			
	a (*n* = 58)	b (*n* = 11)	c (*n* = 52)	d (*n* = 93)
WTP				
Mean	82723 (USD 526)	104537 (USD 665)	80425 (USD 512)	86300 (USD 549)
(JPY)				
SD	12,123	21,714	1,650	13,995
Minimum value	70,699	88,014	78,771	68,674
Maximum value	95,799	136,490	82,420	99,734

*Note:* Group a (those who did not obtain category A/B embryos via PGT‐A), Group b (those who obtained category A/B embryos but were awaiting embryo transfer), Group c (those who transferred category A/B embryos but did not achieve pregnancy or experienced miscarriage), and Group d (those who transferred category A/B embryos and subsequently achieved pregnancy or a live birth). 1 USD = 157.18 JPY (as of December 25, 2024).

Abbreviation: WTP, willingness to pay.

## 4 Discussion

In this study, the mean WTP for an additional embryo submitted for PGT‐A was JPY 84,072 (USD 535) for women and JPY 87,295 (USD 555) for men, with men showing a significantly higher WTP. Both women and men tended to prefer testing plans offered at medical facilities that were closer to home, had more embryologists, and provided longer explanations about PGT‐A and test results. Specifically, a one‐minute increase in the explanation time for the procedure and results were associated with WTP increases of approximately JPY 7000 and JPY 8500, respectively. Although WTP varied by pregnancy outcomes, it exceeded JPY 80,000 even among those who did not achieve a live birth, reflecting a consistently high valuation of PGT‐A.

Economic evaluations of add‐on technologies in ART have gained increasing attention recently. In the United States, PGT‐A is estimated to cost $1150–1650 per embryo [21], which is higher than typical per‐embryo fees in Japan. Despite this high upfront cost, reports indicate that, in selected populations (e.g., women aged ≥ 35 or ≥ 37 years, or those with a history of miscarriage), PGT‐A can reduce the cost per live birth by shortening the IVF treatment period and lowering the miscarriage rate. Furthermore, there is a degree of consensus on the usefulness of PGT‐A in reducing the psychological burden on patients by decreasing the incidence of miscarriage [22]. A survey targeting China found that while the WTP for IVF was USD 4259, the WTP for PGT‐A was USD 6669, equivalent to 0.53 times the national per capita GDP. Additionally, higher educational attainment and receiving cost explanations from physicians were positively correlated with WTP [23]. Our findings similarly suggest that information provision is highly valued, given the substantial WTP for longer explanation time. As mentioned earlier, China has faced rapid population decline in recent years, which may have contributed to a higher perceived value of ART compared to other countries. Although exchange rates and economic conditions vary by country, a similar trend may be observed in Japan as well.

Analysis of WTP using the 2‐class logit model (Table 3) indicated that both women and men placed strong emphasis on the time devoted to explaining PGT‐A and the test results. This preference was stronger among Female‐Class I (preferring small clinics) and Male‐Class I (not concerned about the annual number of PGT‐A performed). Given that women typically make more frequent clinic visits during ART, they may consider nearby small clinics more convenient and better suited to individualized consultations that accommodate personal circumstances. Furthermore, these two groups placed greater value on the number of embryologists at the medical facility than on the facility's annual PGT‐A volume. This may reflect concerns regarding the technical quality and safety of embryo handling.

In this study, the estimated WTP for an additional embryo demonstrated a noticeably narrower range among men (JPY 86,539–87,573) than among women (JPY 72,940–93,333, Table 4). Under the Advanced Medical Care B program, the official out‐of‐pocket costs for PGT‐A were explicitly defined and publicly accessible on the Ministry of Health, Labour and Welfare website, set at JPY 120,400 for the first embryo and JPY 66,400 for each additional embryo. Since the couples in this study had actual experience paying these specified amounts, this official fee structure likely functioned as a baseline reference point. Consequently, men's WTP estimates tightly clustered around a level close to the actual marginal cost. On the other hand, women directly undergo diverse and fluctuating clinical phases, such as oocyte retrieval, embryo transfer, and the confirmation of pregnancy or miscarriage, which entail substantial individual variations in both physical and psychological burdens. Furthermore, in Japan, women frequently play a primary role in managing household finances. Therefore, while women shared the same baseline reference price, their WTP was likely heavily influenced by their direct clinical experiences and financial management perspectives, ultimately leading to the greater variability observed in their valuation.

Notably, both women and men showed a WTP of approximately JPY 6800–7000 for an additional minute of PGT‐A explanation and around JPY 8600 for an additional minute of PGT‐A results explanation. These WTP estimates did not differ statistically between women and men. Compared with increasing laboratory capacity (e.g., annual case volume) or staffing (e.g., the number of embryologists), allocating more time and improving the content of explanation is relatively feasible for individual providers and facilities. Given that PGT‐A remains a substantial add‐on cost, providing thorough explanations to both partners is crucial. The higher WTP for an additional embryo submitted among men also suggests that having both partners present during PGT‐A explanations may help support acceptance and decision‐making at the couple level. The current JSOG guidelines [24] mandate that couples undergoing preimplantation genetic testing receive written explanations about the test's principles, methods, expected outcomes, and safety prior to testing. Because PGT‐A is implemented within infertility treatment primarily provided to women, women are expected to be the insured beneficiaries under public insurance; however, involvement of both partners in counseling should be maintained. A high WTP relative to consultation time may reflect not only patients' demand for information, but also their anxiety regarding the uncertainties of PGT‐A. Providers should prioritize shared decision‐making through meaningful dialogue rather than merely extending one‐way explanations.

Regarding embryo transfer outcomes, Group b showed a significantly higher WTP for an additional embryo submitted, likely reflecting strong expectations for the future transfer of preserved category A/B embryos (Table 5). In addition, Group d (pregnancy or live birth) reported a significantly higher WTP than Group c (no pregnancy or miscarriage), suggesting that favorable outcomes may reinforce the perceived benefit of PGT‐A. Nevertheless, WTP remained substantial even in Group a (no category A/B embryos) and Group c, indicating that patients value not only clinical outcomes but also the information and reassurance provided by PGT‐A. The fact that WTP did not increase further in Group d despite successful outcomes may reflect a ceiling effect under high out‐of‐pocket costs.

In the previous PGT‐A study, the patient burden was JPY 120,400 per embryo (JPY 66,400 for the second and subsequent embryos). This amount was calculated by summing costs such as consumables (reagents), equipment depreciation, and personnel costs for physicians, nurses, and embryologists. Given the design of Japan's healthcare system, it is difficult for patients' WTP to be directly reflected in the setting of medical reimbursement points for new technologies. However, it is important to note that if the assigned reimbursement points do not adequately cover the costs borne by providers and suppliers—including labor, pharmaceuticals, medical devices, and genetic testing technologies—the sustainable provision of PGT‐A may become difficult, potentially harming patients. In addition, given the high value placed on counseling in our DCE, requiring an adequate system for pre‐ and post‐test explanations as a facility criterion for insurance‐covered PGT‐A may be useful; facilities already meeting JSOG requirements are likely to satisfy this criterion. Among the participants in this study, more than half of those who underwent PGT‐A submitted three or more embryos (Table 2), which under the above pricing structure corresponds to an average out‐of‐pocket cost of approximately JPY 80,000–110,000 per embryo, depending on the number submitted. The WTP for an additional embryo submitted derived from this study was approximately JPY 85,000 for both women and men, and there was no marked divergence between WTP and the prices that patients had paid in the previous PGT‐A study. This suggests that, in this setting, the current fee levels are not markedly discordant with patient valuations. However, it is important to note that this amount represents the “willingness”‐to‐pay and not the market price. Under Japan's universal health insurance system, patients generally bear 30% of the total medical costs, with additional expenses exceeding a certain threshold subsidized based on income (the High‐Cost Medical Expense Benefit System). Therefore, if PGT‐A were reimbursed under public insurance, patient acceptance may remain favorable even if the total reimbursement price exceeds the WTP estimated here, because out‐of‐pocket payments would be partially covered and capped. This point is also suggested by the fact that household income did not correlate with WTP in this study.

Regarding WTP survey methods, while the CVM directly asks respondents for the price they are willing to pay, the DCE involves asking them to select the most preferred (or least preferred) set from repeated choice sets. DCE has been increasingly used in recent years due to its ability to model realistic trade‐offs across multiple attributes, including cost [25]. Furthermore, the DCE is well suited to estimating attribute‐level contributions to WTP, which correspond to the secondary outcomes of this study. However, WTP estimates may differ across calculation methods. The greater the difficulty respondents have in distinguishing among options within a choice set, or the greater the variation among options, the larger the discrepancy between WTP estimates calculated using the CVM method and those calculated using the DCE method [26]. In this study, the range of options was set within realistic limits, resulting in a relatively modest variation across options. Consequently, the derived WTP should be interpreted with some uncertainty. Nevertheless, because our objective was to characterize preferences for the PGT‐A service‐delivery system under realistic trade‐offs, a DCE was more suitable than a CVM. Therefore, our results provide a reasonable basis for interpreting patient and partner valuations of PGT‐A in Japan.

## 5 Limitations

Several limitations should be considered when interpreting the findings of this study. First, the choice set options for per‐embryo testing costs were based on a domestic study in 2017–2018 [14], and given recent inflation and yen depreciation, these figures may not accurately reflect current medical costs, potentially leading to an underestimation of price sensitivity in WTP. Second, approximately 75% of participants resided within a 50‐km radius of the study facilities. As some regions in Japan have only a handful of ART facilities within commutable distance, including those populations might result in a nationwide WTP higher than that observed in this study. A further limitation is that our DCE included a predefined set of attributes; facility choice may also be influenced by unmeasured factors such as reputation (word of mouth), appointment availability, and clinic operating hours. Finally, potential selection bias must be noted as the subjects were limited to couples from the previous PGT‐A study. A higher proportion of responding women had actually undergone PGT‐A (95.3%, 204/214) compared to non‐respondents (85.9%, 140/163), suggesting a response bias toward those with favorable impressions of PGT‐A. To accurately understand the values of infertile patients in Japan, future surveys should include couples who are considering PGT‐A but have not yet undergone the procedure, as well as those who considered it but ultimately did not proceed. Conducting a survey targeting couples who did not actually undergo PGT‐A is currently difficult due to the significant effort required for identifying and recruiting eligible patients and other factors. However, such research may be considered if a more detailed evaluation is required in the future.

## 6 Conclusions

Among couples who considered PGT‐A under Japan's Advanced Medical Care B program and participated in the previous PGT‐A study, the mean WTP for an additional embryo submitted for PGT‐A was JPY 84,072 (USD 535) for women and JPY 87,295 (USD 555) for men, exceeding JPY 80,000 even among those who did not achieve live birth. This suggests that couples undergoing PGT‐A value not only favorable outcomes but also the information and the perceived importance of the testing itself. For both women and men, the WTP significantly increased with longer explanations about the PGT‐A test and its results. Since men had a significantly higher WTP for an additional embryo submitted than women, providing couples with sufficient information before and after testing is critically important for improving the acceptability of PGT‐A. These findings are also highly relevant to establishing a sustainable PGT‐A provision system under public health insurance coverage in Japan.

## Funding

This study was funded by Obstetrics and Gynecology, Graduate School of Medicine, the University of Osaka.

## Ethics Statement

The study was approved by the University of Osaka Hospital Observation Research Ethics Review Committee (Approval Number: 24284(T3)).

## Consent

This study was a questionnaire survey, and appropriate consents were obtained from the research subjects. Consents to participate in the study were obtained by checking a box at the beginning of the questionnaire form that was created on the website.

## Conflicts of Interest

The corresponding author (T.T.) is employed by a department funded by Takara Bio, which had no financial involvement in this study. All other authors declare no conflicts of interest.

## Supporting information


**Appendix 1:** Guideline for embryo diagnosis in preimplantation genetic testing formulated by the Japan Society of Obstetrics and Gynecology.

**Appendix 2:** All 10 choice set used in a discrete choice experiment (DCE).

**Appendix 3:** Details of analyzing methods of discrete choice experiment data.

**Appendix 4:** Distribution of respondents by facility.

**Appendix 5:** Infertility treatment history of respondents.

**Appendix 6:** Distance from home to the facility performing PGT‐A.

**Appendix 7:** Estimation results of the conditional logit and random parameter logit models.

## Data Availability

The data that support the findings of this study are available on request from the corresponding author. The data are not publicly available due to privacy or ethical restrictions.

## References

[1] Japan Society of Obstetrics and Gynecology , “ART Data Book,” (2023), https://www.jsog.or.jp/activity/art/2023_JSOG‐ART.pdf.

[2] S. Munné , B. Kaplan , J. L. Frattarelli , et al., “Preimplantation Genetic Testing for Aneuploidy Versus Morphology as Selection Criteria for Single Frozen‐Thawed Embryo Transfer in Good‐Prognosis Patients: A Multicenter Randomized Clinical Trial,” Fertility and Sterility 112, no. 6 (2019): 1071–1079.e7, 10.1016/j.fertnstert.2019.07.1346. 31551155

[3] ESHRE PGT‐SR/PGT‐A Working Group , E. Coonen , C. Rubio , et al., “ESHRE PGT Consortium Good Practice Recommendations for the Detection of Structural and Numerical Chromosomal Aberrations,” Human Reproduction Open 2020, no. 3 (2020): hoaa017, 10.1093/hropen/hoaa017. PMC7257111 32500102

[4] T. Takiuchi , F. Saji , M. Handa , et al., “A Prospective Study on Clinical Utility of Preimplantation Genetic Testing for Aneuploidy and Structural Rearrangement Under Advanced Medical Care B in Japan,” Reproductive Medicine and Biology 25, no. 1 (2026): e70101, 10.1002/rmb2.70101. PMC13643508 42846290

[5] M. García‐Martín , C. Amezcua‐Prieto , B. H. Al Wattar , J. Stener Jørgensen , A. Bueno‐Cavanillas , and K. Saeed Khan , “Patient and Public Involvement in Sexual and Reproductive Health: Time to Properly Integrate Citizen's Input Into Science,” International Journal of Environmental Research and Public Health 17, no. 21 (2020): 8048, 10.3390/ijerph17218048. PMC7663614 33142916

[6] S. Mathur , K. Kirk , C. Dadi , and L. Dougherty , “Women's Involvement in Decision‐Making and Association With Reproductive Health Behaviors: Findings From a Cross‐Sectional Survey in Niger,” BMC Women's Health 24, no. 1 (2024): 278, 10.1186/s12905-024-03115-x. PMC11075281 38715013

[7] C. Skedgel , E. Ralphs , E. Finn , M. Markert , C. Samuelsen , and J. A. Whitty , “How Do People With Experience of Infertility Value Different Aspects of Assistive Reproductive Therapy? Results From a Multi‐Country Discrete Choice Experiment,” Patient 15, no. 4 (2022): 459–472, 10.1007/s40271-021-00563-7. PMC9197909 34940935

[8] F. von Estorff , M. H. Mochtar , V. Lehmann , and M. van Wely , “Driving Factors in Treatment Decision‐Making of Patients Seeking Medical Assistance for Infertility: A Systematic Review,” Human Reproduction Update 30, no. 3 (2024): 341–354, 10.1093/humupd/dmae001. PMC11063545 38305635

[9] R. Cookson , “Willingness to Pay Methods in Health Care: A Sceptical View,” Health Economics 12, no. 11 (2003): 891–894, 10.1002/hec.847. 14601152

[10] C. Steigenberger , M. Flatscher‐Thoeni , U. Siebert , and A. M. Leiter , “Determinants of Willingness to Pay for Health Services: A Systematic Review of Contingent Valuation Studies,” European Journal of Health Economics 23, no. 9 (2022): 1455–1482, 10.1007/s10198-022-01437-x. PMC8853086 35166973

[11] M. V. Bala , J. A. Mauskopf , and L. L. Wood , “Willingness to Pay as a Measure of Health Benefits,” PharmacoEconomics 15, no. 1 (1999): 9–18, 10.2165/00019053-199915010-00002. 10345161

[12] Japan Society of Obstetrics and Gynecology , “The Guideline for Embryo Diagnosis in Preimplantation Genetic Testing,” (2025), https://www.jsog.or.jp/activity/pdf/hai_shindan_shishin.pdf.

[13] D. A. Hensher , J. M. Rose , and W. H. Greene , Applied Choice Analysis: A Primer (Cambridge University Press, 2005).

[14] T. Sato , M. Sugiura‐Ogasawara , F. Ozawa , et al., “Preimplantation Genetic Testing for Aneuploidy: A Comparison of Live Birth Rates in Patients With Recurrent Pregnancy Loss due to Embryonic Aneuploidy or Recurrent Implantation Failure,” Human Reproduction 34, no. 12 (2019): 2340–2348, 10.1093/humrep/dez229. 31811307

[15] Japan Society of Obstetrics and Gynecology , “Report on the Implementation Status of Preimplantation Genetic Testing for Infertility and Recurrent Pregnancy Loss,” (2023), https://www.jsog.or.jp/activity/pdf/2023PGT‐ASRhoukoku.pdf.

[16] H. Shirasawa , M. Yamada , S. C. Jwa , K. Kuroda , M. Harada , and Y. Osuga , “Assessment of Embryologist Sufficiency and Associated Regional Disparities in Japanese Assisted Reproduction Facilities Using Nationwide Survey Data (IZANAMI Project),” Journal of Obstetrics and Gynaecology Research 50, no. 9 (2024): 1459–1469, 10.1111/jog.16022. 38961649

[17] P. C. Boxall and W. L. Adamowicz , “Understanding Heterogeneous Preferences in Random Utility Models: A Latent Class Approach,” Environ Resour Econ (Dordr) 23, no. 4 (2002): 421–446, 10.1023/A:1021351721619.

[18] W. H. Greene and D. A. Hensher , “A Latent Class Model for Discrete Choice Analysis: Contrasts With Mixed Logit,” Transportation Research Part B: Methodological 37, no. 8 (2003): 681–698, 10.1016/S0191-2615(02)00046-2.

[19] J. Shen and T. Saijo , “Does an Energy Efficiency Label Alter Consumers' Purchasing Decisions? A Latent Class Approach Based on a Stated Choice Experiment in Shanghai,” Journal of Environmental Management 90, no. 11 (2009): 3561–3573, 10.1016/j.jenvman.2009.06.010. 19595499

[20] J. Swait , “Advanced Choice Models,” in Valuing Environmental Amenities Using Stated Choice Studies, ed. B. J. Kanninen (Springer, 2007), 229–293, 10.1007/1-4020-5313-4_9.

[21] B. J. Peipert , S. Mebane , M. Edmonds , L. Watch , and T. Jain , “Economics of Fertility Care,” Obstetrics and Gynecology Clinics of North America 50, no. 4 (2023): 721–734, 10.1016/j.ogc.2023.08.002. 37914490

[22] O. Akin‐Olugbade , T. Jain , and A. Komorowski , “A Review of Cost‐Effectiveness of Preimplantation Genetic Testing for Aneuploidy,” 10.1097/OGX.0000000000001373. 40080891

[23] C. Li , H. Cong , S. Jan , L. Si , L. Geng , and S. Li , “Willingness to Pay for Assisted Reproductive Technologies Among Individuals With Infertility in China,” Health Policy and Planning 40, no. 8 (2025): 867–875, 10.1093/heapol/czaf045. PMC12448910 40671365

[24] Japan Society of Obstetrics and Gynecology , Opinion on Preimplantation Genetic Testing for Infertility and Recurrent Pregnancy Loss, https://fa.kyorin.co.jp/jsog/readPDF.php?file=77/8/077081014.pdf#page=27.

[25] V. Soekhai , E. W. de Bekker‐Grob , A. R. Ellis , and C. M. Vass , “Discrete Choice Experiments in Health Economics: Past, Present and Future,” PharmacoEconomics 37, no. 2 (2019): 201–226, 10.1007/s40273-018-0734-2. PMC6386055 30392040

[26] A. Danyliv , M. Pavlova , I. Gryga , and W. Groot , “Willingness to Pay for Physician Services: Comparing Estimates From a Discrete Choice Experiment and Contingent Valuation,” Society and Economy 34, no. 2 (2012): 339–357, 10.1556/socec.34.2012.2.9.

