# Supplementary material for: Patient Willingness‐To‐Pay and Preferences for Preimplantation Genetic Testing for Aneuploidy (PGT‐A) Provision Models in Japan
Source: Reprod Med Biol. 2026 Nov 10;25(1):e70113. doi: 10.1002/rmb2.70113 (PMC13752389; doi:10.1002/rmb2.70113)
Supplement: Supplementary file 1 [file RMB2-25-e70113-s004.docx]

**Appendix 3. Details of analyzing methods of discrete choice experiment data**

In the analysis of discrete choice experiment data, the conditional logit (CL) model is widely used as the baseline specification to investigate how respondents value the presented attributes. In the CL specification, the probability $P_{\mathrm{ij}}$ that individual i selects alternative j from a choice set J is given by

$P_{ij}=\frac{\exp(\beta^{'}X_{ij})}{\sum_{j=1}^{J} \exp(\beta^{'}X_{ij})}$ 　　　　 (1)

where $X_{\mathrm{ij}}$ represents the attributes of the alternative j, and $\beta^{'}$ denotes the parameter vector associated with the attribute matrix $X_{\mathrm{ij}}$.

However, it is extremely difficult to examine heterogeneity among individuals using a CL model. One way to circumvent this difficulty is to use the estimates obtained from the latent class logit (LCL) model. The LCL model assumes that the population consists of several latent classes S and that unobserved heterogeneity among individuals can be captured by these classes by estimating a different parameter vector in the corresponding attribute matrix. Formally, the choice probability of an individual i of class s selecting alternative j is expressed as:

$P_{ij|s}=\frac{\exp(\beta_{s}^{'}X_{ij})}{\sum_{j=1}^{J} \exp(\beta_{s}^{'}X_{ij})}$ , $s=1,...,S$	(2)

where $\beta_{s}^{'}$ is the class-specific parameter vector. Then, in accordance with Boxall and Adamowicz (2002) [16] and Louviere, Hensher and Swait (2000) [17], the probability of an individual i belonging to class s (H_is_) can be expressed as:

$H_{is}=\frac{\exp(\lambda_{s}^{'}Z_{i})}{\sum_{s=1}^{S} \exp(\lambda_{s}^{'}Z_{i})}$	(3)

where $Z_{i}$ denotes a set of characteristics (e.g., individual-specific characteristics) that determine the classification probability, and $\lambda_{s}^{'}$ is the parameter vector in class s associated with $Z_{i}$. Combining Equations (2) and (3), the unconditional probability of choosing alternative j is given as:

$P_{ij}=\sum_{s=1}^{S} P_{ij|s}H_{is}=\sum_{s=1}^{S} \left[ \frac{\exp(\beta_{s}^{'}X_{ij})}{\sum_{j=1}^{J} \exp(\beta_{s}^{'}X_{ij})} \right]\left[ \frac{\exp(\lambda_{s}^{'}Z_{i})}{\sum_{s=1}^{S} \exp(\lambda_{s}^{'}Z_{i})} \right]$	(4)

In Equation (4), the parameter vectors $\beta_{s}^{'}$ and $\lambda_{s}^{'}$ can be simultaneously estimated using the maximum likelihood method to explain the choice behavior.

However, the LCL model cannot be estimated unless S (the number of classes) in Equation (4) is given. Therefore, the central issue in the LCL model is determining S. The literature recommends several information criteria for this purpose [16, 17, 18, 19]. Among these, the Akaike Information Criterion (AIC) and Bayesian Information Criterion (BIC) based on the log likelihood at convergence with s classes, sample size, and number of parameters are popular for determining S.

An alternative approach that can account for individual heterogeneity in discrete choices is called the random parameter logit (RPL) model, which allows the parameters to vary randomly across individuals according to specified distributions (e.g., normal, log-normal, or triangular). Under this framework, each individual is characterized by their own set of parameter values. From this perspective, the RPL model can be viewed as a special case in which every individual in the sample is effectively treated as a separate latent class—corresponding to an LCL model with N classes, where N is the sample size. In contrast, the LCL model accounts for individual heterogeneity by grouping individuals into s classes, where s ranges from 1 to $N$. Compared with the RPL model, a key advantage of the LCL approach is that it is semi-parametric and therefore does not require specific assumptions about the distribution of parameters across individuals [17].

In this study, we estimated the responses to the ten choice sets using the CL, LCL, and RPL models. It should be noted that although the choice sets present “the cost per embryo” as an attribute, in the model estimation this attribute was converted into “the total cost,” calculated as the cost per embryo multiplied by the number of embryos submitted for testing. In a discrete choice experiment, WTP is derived by taking the ratio of an attribute’s marginal utility, measured by its estimated parameter, to the marginal utility of cost, as this reflects the marginal rate of substitution between the attribute and money.　Therefore, the respondents’ WTP for an additional embryo submitted for PGT-A was calculated by the following equation.

$WTP=-\frac{\beta_{number}}{\beta_{total\_cost}}$	(5)

where $\beta_{total\_cost}$ and $\beta_{\mathrm{number}}$ denote the estimated parameters associated with the attributes of "total cost of PGT-A" and "number of embryos submitted for PGT-A", respectively.

In the subgroup analysis, once individual WTP values were obtained, differences in mean WTP between groups were examined using pairwise two-sided t-tests, with the Bonferroni correction applied to adjust for multiple comparisons. In addition, in the analysis of the combined male–female sample, we investigated the extent to which each background characteristic (e.g., education, income, occupation, and number of existing children) contributed to variations in WTP.
